# Genomic characterization of avian pathogenic *Escherichia coli* and its potential as a marker organism for antimicrobial resistance

**DOI:** 10.1128/aem.02244-25

**Published:** 2026-05-04

**Authors:** Pankaj Prakash Gaonkar, Reed Golden, Alinne L. R. Santana-Pereira, Alyssa Lambert, Courtney Higgins, Yagya Adhikari, Matthew Bailey, Kenneth Macklin, Laura Huber

**Affiliations:** 1 Department of Pathobiology, College of Veterinary Medicine, Auburn University 1383, Auburn, Alabama, USA; 2 Department of Poultry Science, College of Agriculture, Auburn University 1383, Auburn, Alabama, USA; 3 Department of Poultry Science, College of Agriculture, Mississippi State University 5547, Starkville, Mississippi, USA; Universidad de los Andes, Bogotá, Colombia

**Keywords:** antimicrobial resistance, poultry, avian pathogenic *E. coli*

## Abstract

Avian pathogenic *Escherichia coli* (APEC) poses a threat to poultry and public health due to its ability to cause avian disease, potentially contribute to foodborne illness, and carry antimicrobial resistance genes (ARGs). Given its ubiquitous nature and capacity to persist in diverse environments, APEC has potential as a marker organism for tracking antimicrobial resistance (AMR) in poultry production systems and surrounding environments. This study investigated the prevalence, genotypic diversity, AMR profile, and potential transmission of APEC across different production stages in commercial, vertically integrated broiler operations. APEC isolates were recovered from environmental samples across the production chain, including pullet, breeder, and broiler farms as well as processing plants. *E. coli* isolates were classified as APEC if carrying three or more out of five virulence genes. Whole genome sequencing was conducted on 42 APECs. We determined their serotypes, phylogroups, sequence types (ST), and AMR profiles and constructed a single-nucleotide polymorphism (SNP)-based phylogenetic tree. High-risk APEC strains were detected, with ST131 recovered at the post-chill stage, raising food safety concerns. All isolates shared a core resistome, while several ARGs were differently distributed across sample types. SNP analysis revealed genetically closely related APEC strains inside the poultry house and the adjacent outside environment, and critically, between broiler litter and carcass rinses collected at the processing plant that processed the same flock. We demonstrate APEC carrying clinically relevant ARGs spread within farm environment and along the production chain, supporting its utility for AMR surveillance.

**IMPORTANCE:**

Avian pathogenic *Escherichia coli* (APEC) is a threat to poultry production and public health due to its ability to cause avian disease, economic losses, potential foodborne implications, and ability to carry antimicrobial resistance genes (ARGs) relevant to human health. In this study, we proposed APEC as a potential marker organism of antimicrobial resistance (AMR) due to its capacity to persist in diverse environments. Our findings show that APEC, across the vertically integrated poultry production chain, harbored clinically important ARGs and exhibited resistance to antimicrobials of human health importance. We also identified evidence of APEC transmission within the farm environment and into the processing plant, with the potential to reach consumers. By examining multiple stages of production and diverse environmental samples, our study provides a more comprehensive understanding of APEC ecology, its resistome, and dissemination. These insights highlight APEC’s utility as a marker organism for AMR surveillance in poultry production.

## INTRODUCTION

*Escherichia coli* has been proposed as an indicator organism for antimicrobial resistance (AMR) surveillance in the environment due to several key advantages (1). AMR profiles of *E. coli* from environmental samples often align with data from human and animal health sectors, facilitating integrated and comprehensive surveillance (1). A key concern is its function as a reservoir of mobile genetic elements that mobilize antimicrobial resistance genes (ARGs) across different ecological environments (2). Therefore, *E. coli* can effectively provide data on AMR and potential risk transmission between humans, animals, and the environment (1, 3). Its ubiquity, high ARG transmission capabilities, and high survival rates make *E. coli* an important tool for tracking AMR transmission across different sectors (1). In the poultry production sectors in the United States, *E. coli* is part of the current surveillance program, with nearly 100% recovery (4).

While *E. coli* is broadly recognized as a key organism for AMR surveillance, focusing specifically on avian pathogenic *Escherichia coli* (APEC) provides additional epidemiological insight. In this study, APEC was defined using a molecular framework based on the presence of virulence-associated genes (VAGs), which is widely applied to identify *E. coli* strains with pathogenic potential in poultry, but it does not confirm phenotypic virulence. APEC represents, in this study, a higher-risk subset of poultry-associated *E. coli* that is frequently linked to both VAGs and clinically relevant ARGs, posing potential risks to animal and public health (5–7). Specifically, APEC has garnered attention due to its association with colibacillosis, a disease responsible for significant economic losses in poultry production worldwide (8). APEC has also been reported to carry ARGs to medically important antimicrobials, increasing its importance as a potential human pathogen (9). While the pathogenic strains often get the attention, recent whole genome sequencing (WGS) studies have highlighted the critical role of nonpathogenic *E. coli* as a reservoir of ARGs and virulence factors (10). Commensal *E. coli* strains from poultry harbor clinically relevant ARGs and may serve as a source of AMR, contributing to the emergence of high-risk strains through horizontal gene transfer (10). Notably, AMR and virulence are not always a linked trait in avian *E. coli*, and ARGs can be prevalent even among non-pathogenic strains (11). This distinction highlights the importance of monitoring AMR beyond clinical cases, especially in healthy birds and their environment across the production chain. In this study, we explore the unique ecological role that molecularly defined APEC can play in tracking AMR dissemination within and across poultry farm settings.

Commercial poultry in the United States is a vertically integrated system (12). In this study, all farms belonged to a vertically integrated broiler production complex, where birds progress sequentially from pullet and breeder farms to hatcheries and broiler farms and then are transported to the processing plants (12). Pullet farms rear young chicks that will later become the breeder flock (12). Breeder farms then provide fertilized eggs to hatcheries, which in turn provide day-old chicks to broiler farms (12). Broilers are grown to market weight before being transported to the processing plant (12). This integrated system has helped in production efficiency (13) and biosecurity (14); however, there are concerns about persistence and transmissions of pathogens across the production chain (15). Tracking the movement of pathogenic and marker organisms throughout the food chain is essential for identifying critical points of AMR transmission in these contexts. However, comprehensive genomic studies that track strains and describe their resistome from healthy birds and farm environment across the vertically integrated poultry production system are lacking. APEC serves as a useful proxy for tracking AMR across poultry production due to its persistence across production stages, carriage of clinically relevant ARGs, and widespread presence in farm and processing plant environments, enabling identification of potential AMR transmission pathways (16–18). However, studies on the movement of APEC and its associated ARGs through the poultry production chain are limited.

Furthermore, AMR bacteria can spread within poultry farm environments, between the indoor facilities and adjacent outdoor areas (19, 20). While previous studies have reported AMR *E. coli* in different poultry farm-associated environmental matrices such as poultry feces, soil, air, and surface water (21), studies on the movement of APEC and its associated ARGs between inside and outside the poultry house and across vertically integrated broiler production chains remain limited. In this study, we address this gap using WGS of environmental APEC recovered from different stages of vertically integrated poultry farms. Our objectives were to (i) describe APEC serotypes, phylogroups, and sequence types (STs); (ii) characterize APEC genotypic AMR profile; (iii) investigate transmission of APEC between the inside poultry house environment and the adjacent outside environment; and (iv) examine the potential for APEC transmission across different stages of poultry production. By uncovering the prevalence of circulating APEC, tracking their genotypes, and describing their AMR profiles in poultry production, we will inform transmission pathways of AMR pathogens across the food chain and points of critical control to mitigate it.

## MATERIALS AND METHODS

### Sampling

Vertically integrated commercial broiler complex belonging to a single company was screened. From this complex, different production stages were included: pullet (*n* = 4), breeder (*n* = 5), and broiler (*n* = 10) farms, along with the processing plant (*n* = 3). Approximately 10% of farm types within the poultry complex were included. All participating farms reported operating under No Antibiotics Important for Human Medicine. For the purpose of this study, all farms were considered as restricted antimicrobial use, because farms reported no antimicrobial use for at least 3 months before sample collection.

A diverse set of samples was screened for APEC prevalence (Table S2), representing both inside and outside environments of poultry farms. Each farm generally had four to eight poultry houses, of which two houses located further apart were chosen for sampling.

The inside environment was defined as areas inside the poultry house where birds are housed and raised. Inside environment samples included litter (*n* = 39), boot swab (*n* = 34), and fan exhaust dust (*n* = 56). Within each house, litter samples were collected as a composite of 10 grab samples taken from different locations across the house to provide a representative pool. For boot swabs, sterile boot swabs (EnviroBootie; Hardy Diagnostics, California, USA) were placed over the disposable boots before entering a poultry house and then walked along the feed and water line, making intentional contact with fresh dropping (22, 23). Fan exhaust dust was collected by swabbing the fan surfaces with sterile 3M sponge stick swabs (3M Food Safety, St. Paul, MN, USA) pre-moistened with buffered peptone water, as described previously (24).

The outside environment was defined as areas immediately surrounding the poultry house. Outside environment samples included soil (*n* = 29), collected from high-traffic areas, such as entryway to poultry house, puddle/drain water (*n* = 58), and fecal samples of non-poultry origin (FSNPOs, *n* = 47) likely originating from wild or domestic animals and birds.

Additionally, three broiler flocks were tracked to the processing plant. Samples were collected from transportation trucks (*n* = 4) used to move the birds, as well as from the processing plant, including carcass rinses from the post-pick (*n* = 32) and post-chill (*n* = 25) stages. Each carcass was placed into a sterile bag containing 400 mL of buffered peptone water, as described previously (24). The post-pick refers to the stage immediately after the feather removal, while post-chill represents one of the final stages of processing, where chicken carcasses are immersed in cold water below 4°C for 3–4 h to inhibit microbial growth (25).

### APEC isolation and characterization

Samples were enriched in buffered peptone water and incubated for 18–24 h at 37°C. After initial enrichment, samples were streaked on McConkey agar and incubated for 18–24 h at 37°C. Following incubation, four suspected *E. coli* colonies were selected per sample for further processing. Colonies were confirmed to be *E. coli* through conventional PCR targeting species-specific gene *ybbW* (26) (Table S1). PCR parameters consisted of an initial denaturation at 94°C for 120 s, followed by denaturation at 94°C for 30 s, annealing at 63°C for 30 s, extension at 68°C for 90 s, for 38 cycles, with a final extension at 72° C for 10 min.

To determine if *E. coli* isolates were APEC, isolates were subjected to multiplex conventional PCR using five virulence-associated genes (VAGs): *iutA*, *iroN*, *hlyf*, *iss*, and *ompT* (27, 28) (Table S1). PCR parameters consisted of an initial denaturation at 94°C for 120 s, followed by denaturation at 94°C for 30 s, annealing at 63°C for 30 s, extension at 68°C for 180 s, for 38 cycles, with a final extension at 72°C for 10 min. The *E. coli* isolates carrying three or more VAGs were considered as APEC in this study (29, 30). Previously characterized APEC isolates (31) were used to standardize the PCR.

Minimum inhibitory concentration of each antibiotic was determined for APEC isolates selected for whole genome sequencing (*n* = 42) using the VITEK 2 system (bioMérieux). The antibiotic panel included ampicillin, amoxicillin–clavulanic acid, cefpodoxime, ceftazidime, imipenem, amikacin, gentamicin, ciprofloxacin, doxycycline, nitrofurantoin, chloramphenicol, and trimethoprim–sulfamethoxazole. *E. coli* ATCC 25922 was used as a quality control strain. Results were interpreted according to Clinical and Laboratory Standards Institute M100 guidelines (32).

### Whole genome sequencing of representative APEC isolates

A total of 42/101 APEC isolates representing each stage of production (pullet [*n* = 2], breeder [*n* = 8], broiler [*n* = 14], transport truck [*n* = 3], processing plant [*n* = 15]) from different sample types (litter [*n* = 11], boot swab [*n* = 8], fan dust [*n* = 2], soil [*n* = 1], FSNPO [*n* = 2], truck [*n* = 3], post-pick [*n* = 7], post-chill [*n* = 8]) were subjected to whole genome sequencing. DNA was extracted from APEC cultures using Qiagen’s DNeasy UltraClean Microbial Kit, following the manufacturer’s instructions. DNA concentration and quality were measured by absorbance ratio at 260/280 and 260/230 using a spectrophotometer. DNA from each sample was paired-end sequenced (NovaSeq PE150, Illumina). The generated reads were quality-checked with FastQC (v0.12.1) (33). The reads were processed, clipped, and trimmed, and short reads were discarded using Trimmomatic (34). Processed reads were *de novo* assembled into contigs using SPAdes (35). Serogrouping was determined using EC typer (36). Multilocus sequence typing was determined using PubMLST with the *E. coli* Achtman scheme (37). Phylogrouping was determined using ClermonTyping (38, 39). ARGs were annotated using the Comprehensive Antibiotic Resistance Database (CARD) (40, 41) with ABRicate (42). CSI Phylogeny (v.1.4) (43) was used to determine the phylogenetic relatedness and construct the single-nucleotide polymorphism (SNP) tree, using APECO2-211 as the reference genome. The resulting phylogenetic SNP-based maximum likelihood tree was visualized using the iTOL platform (44). SNP differences between APEC strains were evaluated using the pairwise SNP difference matrix generated by CSI Phylogeny (v1.4) to assess the potential genetic relatedness among APEC isolates across different farm types and sample types.

## RESULTS

### APEC prevalence

**Fig 1:**
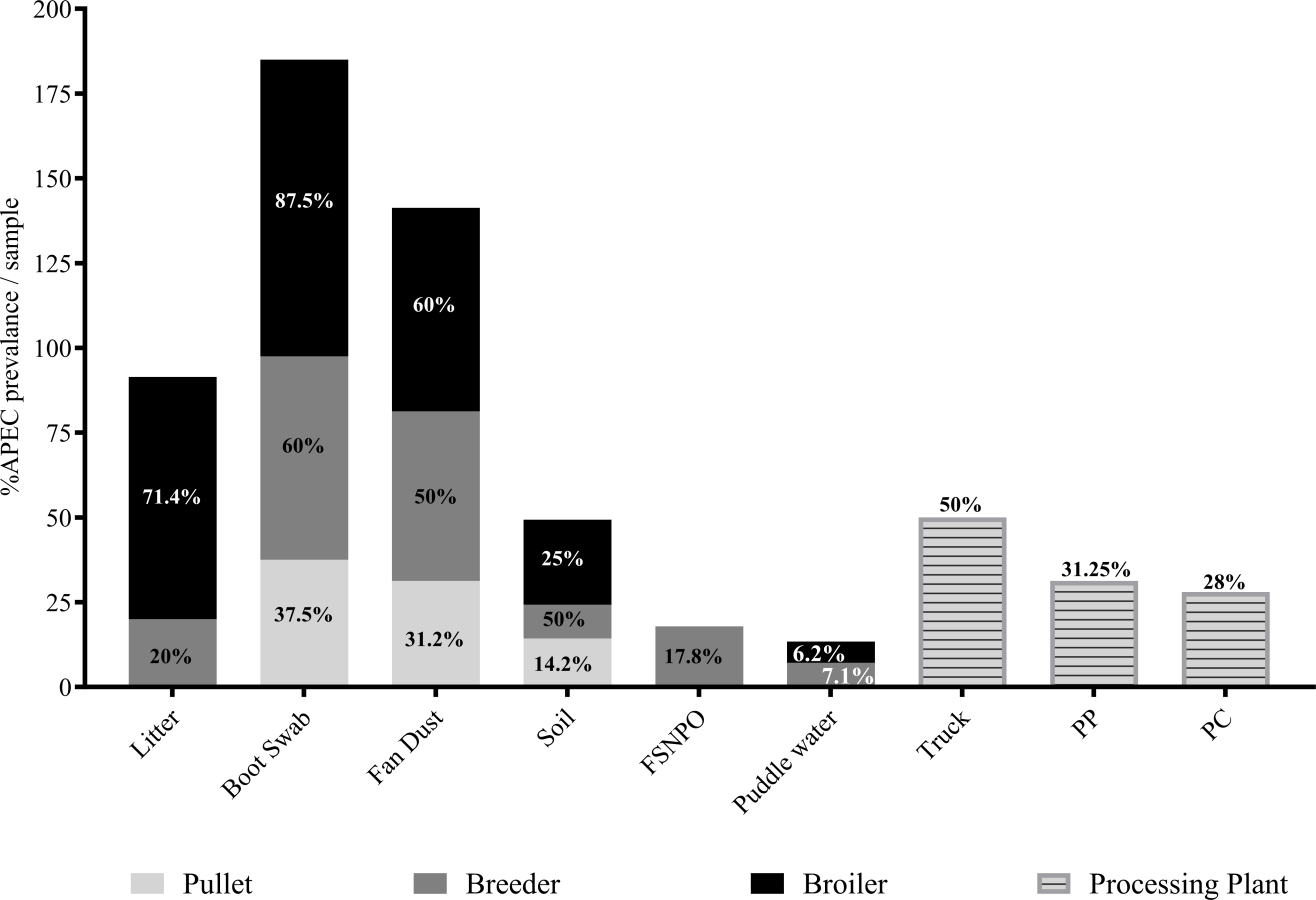
Prevalence of APEC. APEC prevalence across different farm types and sample types. FSNPO, fecal sample of non-poultry origin; PC, post-chill; PP, post-pick.

APEC isolates investigated in this study across different farm types and sample types are shown in Table S2. Among farm types, broiler farms consistently showed higher APEC detection rates (Fig. 1) across multiple sample types compared to pullet and breeder farms. Overall, the highest APEC prevalence was observed in broiler farm litter samples (71.43%), boots swabs (87.5%), fan dust (60%), and soil samples (25%). FSNPO (17.85%) and puddle water (7.14%) samples had the highest prevalence of APEC in breeder farms compared with other farm types. APEC prevalence was lower in pullet farms compared with other farm types for most sample types, except for soil, where APEC prevalence was higher in pullet farms (14.28%) than in breeder farms (10%). Within the processing plant, APEC was detected in 50% of transport truck samples, 31.25% of post-pick samples, and 28% of post-chill samples (Fig. 1).

Prevalence of APEC was also investigated relative to the total *E. coli* detected in each sample and farm type (Fig. S2). APEC prevalence relative to *E. coli* was highest in broiler farms (43.13%), particularly in boot swab (63.54%), soil (47.08%), and fan dust (43.18%) samples. The highest APEC prevalence in FSNPOs was in breeder farms (16.66%) compared with other farm types. Relative APEC prevalence in pullet farms was lower overall, except in soil samples, where it was higher (16.66%) than in soil samples from breeder farms (7.14%). In the processing plant, the relative APEC prevalence was 39.99% in transport trucks, 42.78% in post-pick, and 45.83% in post-chill samples.

Together, these results indicate that broiler farms not only had a higher frequency of APEC-positive samples (51/104) but also had the highest proportion of APEC among *E. coli* (43.13%), followed by breeder (21/39, 19.14%) and pullet (9/63, 9.78%) farms (Fig. 1; Fig. S2).

For further characterization, a subset of 42 APEC isolates was subjected to WGS. These included 2 APEC isolates from pullet farms, 8 from breeder farms, 14 from broiler farms, 3 from transport trucks, and 15 from the processing plant.

### Characterization of APEC isolates

APEC isolates collected across different farm types and sample types exhibited considerable serogroup diversity. Serogroups were distributed across all farm types and samples. The most frequently observed serogroups included 025:H4 (detected in pullet fan dust, broiler litter samples, and post-chill samples), 08:H25 (detected in post-pick and post-chill samples), and 081:H7 (detected in broiler litter and post-pick and post-chill samples).

Overall, 23 different STs were detected among APEC isolates, with one isolate remaining unclassified. The most frequently detected STs were ST117, ST155, and ST58. ST117 was detected in pullet fan dust, breeder litter, broiler litter, and broiler boot swab samples. ST155 was detected in FSNPO surrounding breeder farms, broiler soil, broiler litter, and transport truck. ST58 was detected in breeder litter and post-pick and post-chill samples from processing plants.

APEC isolates in this study were classified into eight different phylogroups, while three isolates remained unclassified. The most frequent phylogroup was B1, detected in 18 different isolates. APEC isolates from pullet farms were classified into phylogroups B2 and G. Breeder farm APEC isolates were classified into A (boot swab), B1 (boot swab, litter, and FSNPO), and G (litter). Broiler farms exhibited the highest phylogroup diversity, including A, B1, B2, C, D, F, and G. Litter samples from broiler farms contained isolates belonging to phylogroup A, B1, B2, D, F, and G. Boot swab isolates included phylogroups A, C, F, and G. In transport truck samples, phylogroups A, B1, and D were detected. At the processing plant, B1 was detected in both post-pick and post-chill samples, B2 and F in post-chill, and E in post-pick samples.

### Phenotypic AMR profile

Overall, 38.1% (16/42) of isolates were not susceptible (resistant or intermediate) to at least one antimicrobial tested (Table S4). In addition, 7.1% (3/42) of isolates were classified as multidrug resistant (MDR), defined as resistance to three or more antimicrobial classes (45).

At pullet farms, no resistance was detected (0/2). At the breeder farm, the prevalence of isolates that were resistant to doxycycline was highest (37.5%, 3/8), followed by ampicillin (25.0%, 2/8), cefpodoxime (12.5%, 1/8), and trimethoprim–sulfamethoxazole (12.5%, 1/8). In broiler farms, resistance was detected for doxycycline (14.3%, 2/14), while chloramphenicol (7.1%, 1/14) showed intermediate resistance.

In the farm environment, resistance was detected only in the inside environment samples (litter and boot swabs). The most frequent was resistance to doxycycline (23.8%, 5/21), followed by ampicillin (9.5%, 2/21), cefpodoxime (4.8%, 1/21), and trimethoprim–sulfamethoxazole (4.8%, 1/21). Intermediate resistance to chloramphenicol (4.8%, 1/21) was also recorded. No resistance was observed in the outside environment (0/3).

In truck samples, resistance was observed for ampicillin (33.3%, 1/3), doxycycline (33.3%, 1/3), and nitrofurantoin (33.3%, 1/3), with intermediate resistance to ciprofloxacin (33.3%, 1/3).

At the processing plant, resistance patterns varied by stage. In the post-chill stage, two isolates exhibited intermediate resistance to chloramphenicol (25.0%, 2/8). In the post-pick stage, prevalence of resistant isolates was highest for doxycycline (42.9%, 3/7), followed by ampicillin (14.3%, 1/7), amoxicillin–clavulanic acid (14.3%, 1/7), cefpodoxime (14.3%, 1/7), ceftazidime (14.3%, 1/7), and nitrofurantoin (14.3%, 1/7). Intermediate resistance was also noted for chloramphenicol (14.3%, 1/7).

### Genotypic resistome

#### Prevalence of ARGs in APEC across different production stages

A comprehensive analysis of 42 APEC isolates from different stages of vertically integrated poultry production revealed the presence of ARGs (Table S3, Fig. S1) associated with AMR to multiple antimicrobial classes, including aminoglycoside (*n* = 6 genes), aminocoumarin (*n* = 3 genes), bacitracin (*n* = 1 gene), β-lactam (*n =* 6 genes), diaminopyrimidine (*n* = 1 gene), fluoroquinolone (*n* = 4 genes), fosfomycin (*n* = 1 gene), macrolide (*n* = 1 gene), nitroimidazole (*n* = 1 gene), polymyxin (*n =* 2 genes), peptide (*n* = 2 genes), sulfonamide (*n* = 1 gene), tetracycline (*n* = 4 genes), and multidrug resistance (*n* = 24 genes). Among these, MDR-associated ARGs were the most prevalent, with their detection in all APEC isolates across different production stages.

### Distribution of non-core ARGs in APEC

**Fig 2:**
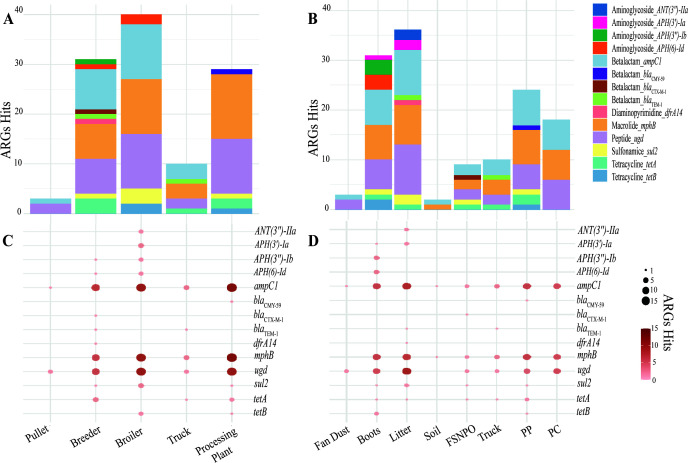
Distribution of non-core ARGs detected across environmental sample types in the production chain. ARG frequency is based on the CARD database annotation. The stacked bar plot shows the total number of non-core ARG hits, categorized by gene, across (**A**) production stages and (**B**) different sample types. The balloon plot depicts the frequency of non-core ARGs across the (**C**) production stages and (**D**) sample types. Balloon size and red color intensity correspond to non-core ARG frequency. FSNPO, fecal sample of non-poultry origin; PC, post-chill; PP, post-pick.

In this study, we considered ARGs that are present in 90% or more APEC isolates tested as core ARGs (46, 47), whereas less than 90% were considered non-core ARGs. From 57 ARGs annotated using the CARD database, 43 were core, and 14 were considered non-core. Although many ARGs were considered core genes in this study (*n* = 43, 75.43%) (Fig. S1), certain ARGs were distributed differently (*n* = 14, 24.56%) in sample types and production stages (Fig. 2A through D). Among aminoglycoside-associated ARGs, four ARGs showed limited presence. *ANT(3″)-IIa* was detected in only two APEC isolates, both from broiler litter samples, suggesting farm-specific occurrence rather than widespread presence. Similarly, *APH(3′)-Ib* was detected in three APEC isolates, one from a boot swab from a breeder farm and two from boot swabs from a broiler farm. *APH(3′)-Ia* was found in three APEC isolates, including litter and boot swabs from the same broiler farm, and in litter from a different broiler farm. Additionally, *APH(6)-Id* was detected in boot swabs from one breeder farm and two broiler farms. Thus, the stage of production may have an influence on the persistence of specific ARGs.

Among β-lactam-associated ARGs, three out of six were distributed differently across the production stages. Notably, *bla*_CMY-59_ was identified exclusively in a single APEC isolate at the post-pick stage of the processing plant, indicating possible association with the carcass processing stages rather than farm-level presence. *bla*_CTX-M-1_ was found only in the FSNPO surrounding a breeder farm, highlighting the potential role of wildlife or free-roaming animals and outside environment in disseminating clinically relevant ARGs. Furthermore, *bla*_TEM-1_ was identified in APEC isolates from both breeder litter and transportation truck samples.

The presence of *dfrA14*, a diaminopyrimidine-associated ARG, was limited to a single APEC isolate from a breeder litter sample, suggesting either a low prevalence within poultry environment or a farm-specific acquisition.

A macrolide-associated ARG, *mphB*, was found in 33 out of 42 APEC isolates. This ARG was absent in APEC isolates from pullet farm samples.

A sulfonamide-associated ARG, *sul2*, was detected in five APEC isolates, originating from FSNPO surrounding a breeder farm, a boot swab, two litter samples from broiler farms, and one sample from the post-pick stage in the processing plant.

A tetracycline-associated ARG, *tetA*, was detected in six APEC isolates, spanning multiple production environments. *TetA* was detected in APEC isolates from FSNPO near breeder farms, in breeder litter samples, and boot swab samples from three distinct breeder farms. *TetA* was also detected in the APEC isolates from transportation truck and two post-pick samples. Furthermore, *tetB* was detected in three APEC isolates, including boot swabs at two different broiler farms and one post-pick sample. The widespread presence of this ARG may indicate common selective pressure across the production chain.

Polymyxin and peptide-associated ARGs were detected in all APEC isolates. However, *ugd* was found in 33 out of 42 APEC isolates, indicating a lower prevalence compared to other polymyxin and peptide ARGs. Finally, ARGs associated with aminocoumarin, bacitracin, fluoroquinolone, fosfomycin, nitroimidazole, and multidrug resistance were universally detected across all APEC isolates. The ubiquitous presence of ARGs across poultry production stages may reflect the prolonged environmental persistence or intrinsic AMR within these APEC isolates.

### Phylogeny of APEC across the production chain

**Fig 3:**
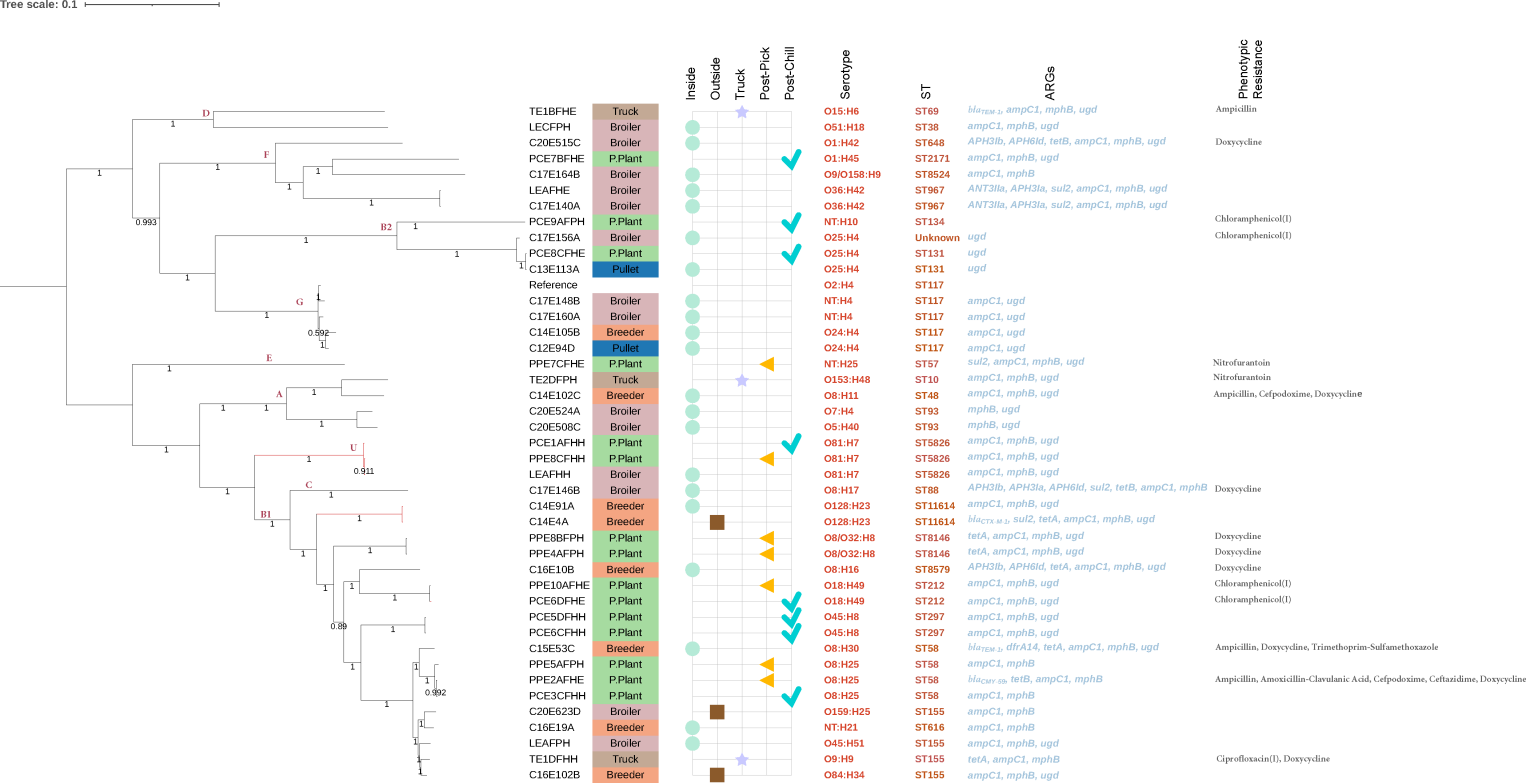
SNP-based phylogenetic tree of APEC isolates. A maximum likelihood phylogenetic tree was generated using CSI phylogeny 1.4, using APEC02-211 from GenBank as the reference genome. The tree was mid-point rooted for visualization using iTOL. Cluster labels denote the phylogroups corresponding to each isolate within the respective clades. Farm type, sample type, serogroup, sequence type (ST), non-core ARGs, and phenotypic resistance profile associated with each isolate are annotated alongside the tree. Red-colored nodes indicate the genetically closely related APEC strains based on SNP differences (SNP < 20). ARG, antimicrobial resistance gene; FSNPO, fecal sample of non-poultry origin; I, intermediate resistance; NT, not typed.

An SNP-based phylogenetic tree was constructed using 42 APEC isolates recovered in this study (Fig. 3). Overall, APEC isolates presented considerable genetic variability, with no consistent clustering patterns within farm type or sample type. These isolates were grouped into eight clusters corresponding to defined APEC phylogroups A, B1, B2, C, D, F, G, and E, as previously described (38, 39). There was one APEC cluster with an unclassified phylogroup (U).

Analysis of SNP distance matrix identified four genetically closely related APEC, defined by pairwise SNP difference of less than 20 SNPs (Fig. 3). Notably, genetically closely related APEC (SNP ≤ 14) were detected from a broiler farm litter sample and processing plant samples (post-pick and post-chill) originating from the same farm and flock, suggesting potential transmission of APEC isolates from the broiler farm to the final stages of carcass processing (post-chill). Additionally, genetically closely related APECs (SNP = 17) were detected from breeder farm litter and from FSNPO, both collected from the same farm, indicating potential transmission between inside the poultry house (litter) and the farm’s surrounding environment (FSNPO).

## DISCUSSION

### Emergence of APEC upstream of the production chain

Our study demonstrates that APEC can be detected at upstream of the vertically integrated poultry production cycle, i.e., pullet and breeder farms. Birds in the pullet stage are 0–21 weeks old and are moved into the breeder farms where they may stay until 68 weeks. To our knowledge, there are no reported cases of APEC detection in pullet farms within the broiler production complex. Existing reports from upstream of the production chain primarily focus on breeder farms in the broiler industry. Previous reports from breeder farms in the southeastern United States (Mississippi) showed B2 phylogroup dominance, followed by D and B1 (31), whereas in another study from the southeastern United States (Georgia), the most prevalent phylogroups were B2, G, and A (48). In contrast to previous reports, our study showed a higher prevalence of phylogroup B1, which is often associated with the commensal strains with less virulence potential but that have been isolated from yolk sac infection cases (11). Discrepancy with our studies may stem from the fact that our samples were collected from environmental sources rather than clinical cases, potentially influencing APEC strain distributions. The detection of APEC at an upstream stage is concerning, as it raises the risk of vertical transmission through eggs, potentially infecting chicks. This mode of transmission from breeders is well documented and can contribute to early chick mortality in broiler flocks (49). Understanding the origin, prevalence, and resistome of APEC isolates is crucial for improving disease prevention in the later stages of broiler production. It is also important to investigate the spread of multidrug-resistant APEC, as they serve as a reservoir of ARGs. Early detection at the pullet and breeder stages may play a key role in reducing both the APEC burden and the dissemination of ARGs throughout the production chain.

### Spread of high-risk APEC strains across the production chain and public health implications

A key finding of this study was the detection of high-risk APEC serotypes ST117 and ST131 (50) across the different stages of production. ST117 is a well-known emergent poultry-associated pathogen (6) that is highly virulent with zoonotic potential (6, 51), whereas ST131 is a foodborne uropathogen previously detected in poultry products (7). Notably, in our study, ST117 (O24:H4, phylogroup G) and ST131 (O25:H4, phylogroup B2) were detected in the exhaust fan dust present inside the poultry house from pullet farms. The presence of these strains in the fan dust is noteworthy, as it suggests the possibility of airborne dissemination of high-risk APEC. Furthermore, we found ST117 (phylogroup G) from a litter sample in one breeder farm. The presence of high-risk APEC at the initial stages of production suggests that these stages might serve as a reservoir for APEC, highlighting the importance of early detection of APEC before reaching consumer-ready products.

In addition, APEC ST117 is also commonly linked to colibacillosis, together with other STs found in our study in later stages of production (broiler farms), including ST88, ST155, ST38, ST648, and ST93 (52, 53). High-risk APEC ST117 was present in two broiler farms in litter and boot swab samples, highlighting that ST117 occurs early in the birds’ life, persisting to later stages. Notably, all ST117 APEC strains belonged to phylogroup G, indicating the presence of high-risk, MDR APEC strains across these farm environments. Phylogroup G has been associated with highly virulent, antimicrobial-resistant *E. coli* (39, 48). A prior study from the southeastern United States (Georgia) found that APEC isolated from colibacillosis spanned across all the phylogroups, but B2, F, and G phylogroups were mostly prevalent in broilers (48).

The presence of APEC in the processing plant carries significant public health risk. APEC has long been considered in the context of animal diseases; however, there is a growing recognition regarding genomic overlap between APEC and extraintestinal pathogenic *E. coli* (ExPEC) (54). APEC and human uropathogenic *E. coli* (UPEC) have been reported to share VAGs, suggesting that APEC may act as a potential reservoir of virulence determinants relevant to human UPEC (55). In addition to APEC prevalence in poultry farms, several reports have identified APEC in retail meat, particularly chicken, which has been recognized as a major source of pathogenic *E. coli* linked to human urinary tract infections (17). A recent study in the United States reported the presence of antimicrobial-resistant *E. coli* in retail chicken meat, conferring resistance to aminoglycosides, β-lactams, folate pathway antagonists, quinolones, and tetracycline (56). Additionally, APEC has been identified in retail chicken meat (18), raising potential concerns about the zoonotic transmission of antimicrobial-resistant APEC and the dissemination of ARGs through the food chain.

In our study, 15 APEC isolates recovered from processing plants were investigated. Most isolates belonged to phylogroup B1, but there was variation in their respective serogroups and STs. The detection of ST131 (025:H4, phylogroup B2) at the post-chill stage is noteworthy because ST131 is a high-risk ExPEC lineage with zoonotic potential. In previous reports, ST131 lineages have been detected in retail poultry meat and associated with their persistence in processing plants (7). Moreover, certain APEC strains are potential foodborne pathogens; e.g., strain ST131:H22 is capable of infecting humans as well as poultry (7). Interestingly, ST131 was found only in the pullet farm and post-chill stage. In the processing plant, its presence in the post-chill stage but absence at the earlier post-pick stage suggests that certain APEC serotypes might not originate from the farm but from the processing plant environment. The detection of ST131 in a post-chill stage in our study strengthens the concern that certain APEC strains can withstand the processing interventions or may be introduced during the post-chill stage, potentially reaching the consumers.

The public health implications of antimicrobial-resistant APEC are concerning, as APEC strains have been linked to human infection and transmission of AMR, especially via consumption of contaminated poultry products (7, 57). A case–control study reported that people infected with antimicrobial-resistant *E. coli* causing urinary tract infections were associated with frequent consumption of chickens (57). All APEC isolates from the processing plant carried multiple ARGs against medically important antimicrobials (e.g., sulfonamide, β-lactams, and tetracyclines). While the precise entry point of APEC into the food chain remains unclear, its presence in poultry processing plants and retail chicken meat (18) raises concerns about potential transmission pathways.

### APEC resistome profile reveals spread of ARGs across the production chain

Globally, studies have reported APEC isolates exhibiting AMR towards a broad range of antimicrobials, including colistin, sulfonamides, florfenicol, amoxicillin, tetracycline, doxycycline, apramycin, neomycin, flumequine, cotrimoxazole, spectinomycin, lincospectin, and cephalosporins (16). ARGs reported in APEC include *mcr-1*, *bla*_CTX-M_, *bla*_CMY-59_, *bla*_TEM_, bla_OXA_, *floR*, *tetA*, *tetB*, *tetC*, *tetD*, *tetE*, *tetG*, *sul1*, *sul2*, *aadA1*, *aadA2*, *cmlA*, *cat1*, *cat2*, and *cat3* (16). Furthermore, a recent meta-analysis revealed a diverse resistome in APEC across the globe (53). AMR was detected against penicillins, pleuromutilins, lincosamides, macrolides, antifolates, sulfonamides, cephalosporins, bacitracins, fluoroquinolones, tetracyclines, quinolones, aminocyclitols, aminoglycosides, chloramphenicols, phosphonic acids, nitrofurans, polymyxins, carbapenems, and monobactams (53). Notably, colistin-resistant APEC has been detected in seven countries but not in the United States (53). Overall, these global reports highlight APEC’s potential as an MDR pathogen and reservoir for AMR to medically important antimicrobials.

In our study across the poultry production chain, we detected the ARGs known to confer AMR to medically important antimicrobials (58). All APEC isolates consistently carried a resistome of 36 ARGs considered the core resistome of the isolates in our study, regardless of sample type or production stage. The core ARGs represent the inherent AMR potential of APEC, enabling its survival under various environmental pressures in poultry farms. Beyond the core ARGs, we identified 14 ARGs which were variably distributed across different production stages (Fig. 2A and C) and sample types (Fig. 2B and D). In this study, we place emphasis on these non-core ARGs due to their potential role in shaping AMR patterns.

A recent study (59) from the southeastern United States reported widespread AMR in APEC isolates to penicillin, clindamycin, erythromycin, and tylosin tartrate, alongside a higher prevalence of ARGs, such as *emrE*, *emrK*, *emrY*, *evgA*, and *evgS*. Our study found similar patterns with *emrK* and *emrY* detected in all 42 APEC isolates, while *evgA* and *evgS* were detected in 40 and *emrE* in 31 APEC isolates. Additionally, limited sulfonamide resistance was reported (59), with *sul2* being detected in 4 out of 10 APEC isolates. Similarly, in our study, we observed low *sul2* prevalence with its detection in only five APEC isolates. However, unlike previously reported (59), we did not detect *sul1* in any of our APEC isolates. Furthermore, in the same report (59), investigators found *AAC(3)-ID*, *AAC(3)-IV*, *AAC(3)-Iva*, *aadA*, *ANT(3″)-IIa*, *APH(3″)-Ib*, *APH(3″)-Ia*, *APH(4″)-Ia*, and *APH(6″)-Id* associated with aminoglycoside resistance in APEC isolates. In our study, while we detected *ANT(3″)-IIa*, *APH(3')-Ia, APH(3')-Ib, APH(6)-Id* in less than three APEC isolates, *acrD* and *kdpE* were present in all our APEC isolates. Since the previous study (59) was conducted in the southeastern US region, geographically closer to our study area, the similarities for some ARGs suggest the persistence of certain AMR determinants in the poultry environment, while the observed differences may reflect regional or environmental influences affecting the ARGs distribution.

A study (31) on a broiler breeder farm in the southeastern United States reported *tetA* and *APH(3″)-Ia* as the most prevalent ARGs, followed by *aadA*, *qacEd*, *bla*_TEM_, *bla*_CTX-M_, and *sul1*. In contrast, our study detected *tetA*, *APH(3″)-Ia*, *bla*_CTX-M-1_, and *bla*_TEM-1_ but in fewer APEC isolates. Interestingly, *tetA* was present in a limited number in our study but detected across multiple stages, including breeder, broiler, transport truck, and processing plant. Furthermore, *APH(3″)-Ia* was detected only in broiler farms, while *bla*_TEM-1_ was detected only in breeder farms. Interestingly, *bla*_CTX-M-1_ was identified in FSNPO surrounding a breeder farm, suggesting wild or domestic free-roaming animals as a potential source of ARG dissemination. A comparison with our study suggests that while breeder farms are key sites for certain ARGs, some ARGs (Fig. 2A and C) do not remain confined to breeder stage and can be found in more advanced stages of the production chain.

In this study, production stage-specific resistome dynamics was observed where APEC from broiler farms had higher ARG diversity and frequency compared to upstream production stages. The stage of production might have an influence on the development of specific AMR. For example, *ANT(3″)-IIa* was detected in only two isolates, both originating from broiler litter samples. *bla*_CTX-M-1_ was detected in an APEC from FSNPO and *dfrA14* in APEC from litter, both originating from breeder farms environment. The variable distribution of APECs carrying certain ARGs might be due to differences in antimicrobial exposure, biosecurity practices, or environmental contamination across stages of production.

Furthermore, antimicrobial-resistant APECs were not confined to the farm environment but were also detected in the processing plant samples, indicating potential for transmission across the production continuum and carryover from a previous batch of processed chickens. APEC isolates from the processing plant carried multiple ARGs against important antimicrobials. Specifically, *sul2*, *bla*_CMY-59_, and *tetB* were detected in post-pick samples, while *tetA* and *ugd* were detected in post-pick and post-chill samples. Notably, *bla*_CMY-59_ was identified exclusively in the post-pick stage of processing plants, indicating a possible association with contamination at the processing plant rather than at the farm level.

Comparison of phenotypic and genotypic resistance was in partial agreement. The presence of ARGs did not always correspond to phenotypic resistance (Fig. 3), although certain ARGs for ß-lactams (*ampC1*, *bla*_TEM-1_, and *bla*_CMY-59_), tetracycline (*tetA* and *tetB*), and trimethoprim (*dfrA14*) were frequently associated with their respective phenotypic profiles. In some isolates, ARGs were detected without a corresponding phenotypic resistance, likely due to silent ARGs (60) and/or associated fitness cost (61). In contrast, phenotypic resistance was observed in some isolates without detectable corresponding ARGs (e.g., cephalosporins, chloramphenicol, ciprofloxacin, and nitrofurantoin). Such cases may be associated with efflux pump activity (62), chromosomal mutations contributing to AMR or ARGs not captured by the current database (63). Discrepancies between genotypic and phenotypic resistance have been documented, often resulting from gene silencing, regulatory effects, or adaptive resistance (64). These findings emphasize the need to integrate genotypic and phenotypic analyses combined to more accurately capture the AMR landscape. Our APEC isolates originated from environmental samples rather than clinical cases. The limited phenotypic resistance observed may be due to the environmental origin of isolates tested, where ARGs often remain silent (60, 65).

### Tracking APEC: evidence of dissemination from farm to processing plant and between farm environments

By analyzing the SNP differences, we identified possible APEC transmission from broiler farms to the processing plant. We defined genetically closely related APEC as isolates with SNP differences of less than 20. In one broiler farm, an ST5826 (O81:H7, phylogroup unknown) isolate from litter was genetically closely related (SNP ≤ 14) to those recovered from post-pick and post-chill samples from the same flock, sharing a similar AMR profile. This specific finding strongly indicates that a particular APEC strain ST5826 (O81:H7, phylogroup unknown) was disseminated from the broiler farm to the final stage of the processing plant. This continuity indicates that the transmission of APEC along the production chain to consumer-ready products is possible.

Furthermore, we were interested in looking at any event of APEC transmission between the inside poultry house to the outside environment. Notably, in one breeder farm, we found that boot swab samples inside the house and the FSNPO from the vicinity shared a genetically closely related (SNP = 17) APEC strain ST11614 (O128:H23, phylogroup B1). These APEC isolates from FSNPO carried additional ARGs, including *bla*_CTX-M-1_, *sul2*, and *tetA*, indicating wild or domestic free-roaming birds/animals may act as external reservoirs and be capable of introducing AMR into nearby poultry farms. Previous reports have suggested that wild birds such as pigeons can act as a reservoir for APEC (66).

### Conclusion

The findings from our study provide epidemiological insights into the prevalence, genetic diversity, and dissemination of APEC and its ARGs across the vertically integrated poultry production system. Our findings highlight the potential of APEC to be utilized as a marker organism for AMR surveillance in poultry. Its ability to harbor ARGs associated with medically important antimicrobials to humans, potential zoonotic risk, and transmission across the production stages makes it a robust marker for monitoring AMR across poultry systems. Notably, a high-risk strain (ST117, phylogroup G) carrying multiple ARGs was consistently found in different stages of poultry production, spanning from pullet to breeder to broiler farms. Furthermore, the detection of a ST131 strain, a potential foodborne uropathogen (7), exclusively in post-chill stage suggests possible introduction at processing plant and raises concerns about foodborne potential originating from cross-contamination and hygiene practices at this stage. The consistent presence of core ARGs across all APEC isolates, regardless of production stage or sample type, highlights the environmental persistence of MDR APEC. Certain APEC strains carrying specific ARGs were detected at some stages of the poultry production chain but were absent at others. This differential distribution of APEC harboring non-core ARGs across different production stages suggests that the farm environment may play a significant role in the persistence and maintenance of ARGs. Factors such as historical antimicrobial use, environmental contamination, and microbial interactions within each stage may influence which ARGs are retained or lost.

An important limitation of our study is the relatively small sample size for the number of APEC isolates tested. Nevertheless, we provide a genomic assessment of APEC across the vertically integrated poultry production chain, rather than focusing on a single production stage. Our APEC isolates were recovered from environmental samples rather than clinical cases; thus, their pathogenic potential remains unknown. Importantly, the presence of VAGs alone does not equate to pathogenicity. Such environmental isolates are less frequently studied but offer a critical insight into the AMR reservoir potential of APEC in the poultry production environment. Continued environmental surveillance is essential to better understand APEC ecology and to utilize APEC as a marker organism for monitoring AMR trends and guiding AMR mitigation strategies within poultry production systems.

## Data Availability

All Illumina reads generated in this study are deposited in the NCBI SRA database (BioProject PRJNA1348499).

## SUPPLEMENTAL MATERIAL

Figures S1 and S2 aem.02244-25-s0001.docx Distribution of ARGs across APEC isolates and prevalence of APEC relative to *E. coli*.

Tables S1 to S5 aem.02244-25-s0002.xlsx Primer details, APEC prevalence, APEC characteristics, and SNP matrix.

ASM does not own the copyrights to Supplemental Material that may be linked to, or accessed through, an article. The authors have granted ASM a non-exclusive, world-wide license to publish the Supplemental Material files. Please contact the corresponding author directly for reuse.
