# Supplementary material for: Genomic characterization of avian pathogenic Escherichia coli and its potential as a marker organism for antimicrobial resistance
Source: Appl Environ Microbiol. 2026 May 4;92(5):e02244-25. doi: 10.1128/aem.02244-25 (PMC13188900; doi:10.1128/aem.02244-25)
Supplement: Figures S1 and S2 — Distribution of ARGs across APEC isolates and prevalence of APEC relative to E. coli. [file aem.02244-25-s0001.docx]

**
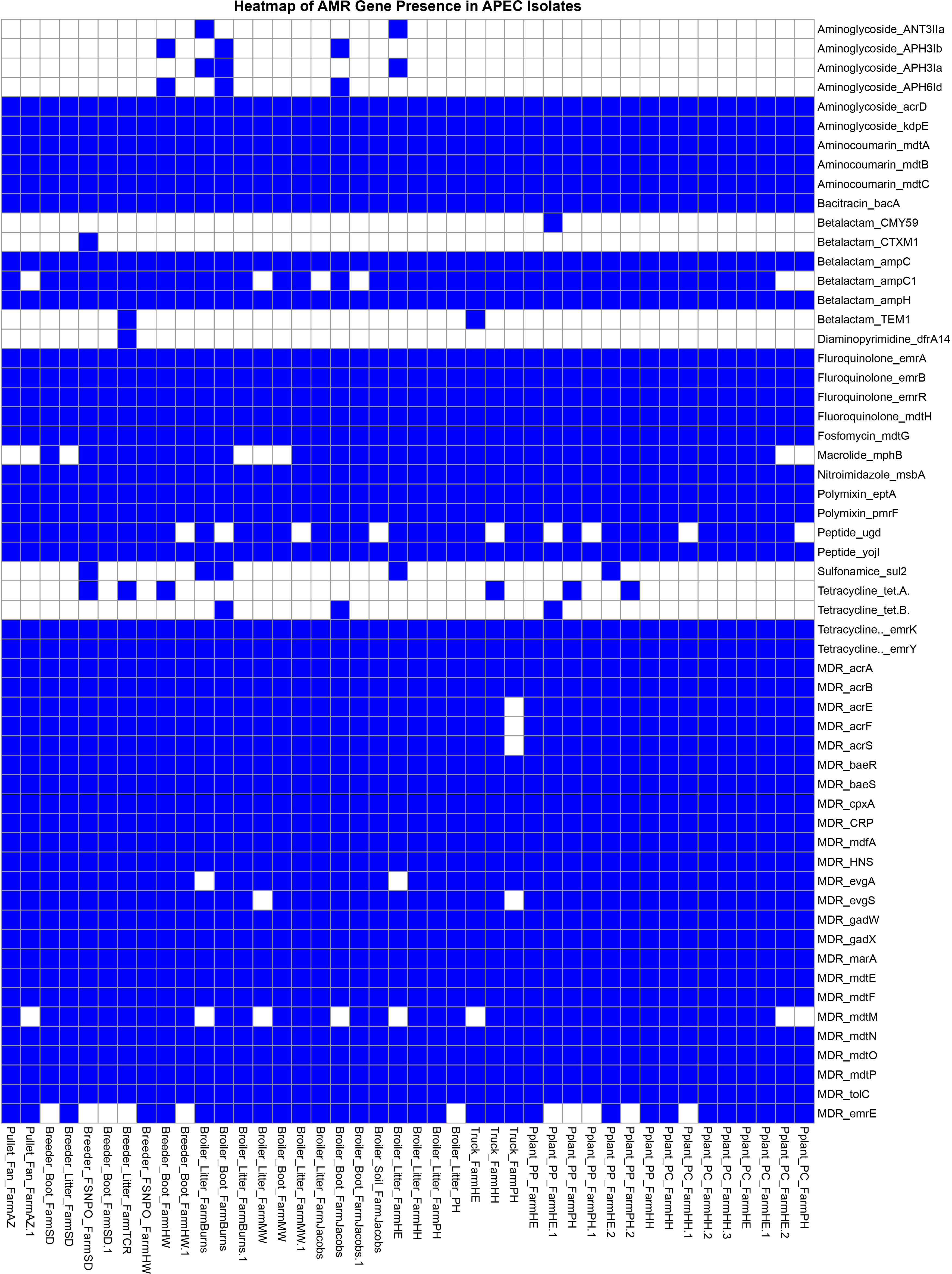
**

**Supplementary Figure 1.** Heatmap showing the distribution of ARGs across APEC isolates. ARGs frequency based on the CARD database annotation. Many ARGs were present in more than 90% of APEC isolates and are considered part of the core resistome in this study. Certain ARGs were found in less than 90% of APEC isolates, and in this study, were considered as the non-core ARGs. FSNPO: fecal samples of non-poultry origin, PP: post-pick, PC: post-chill.


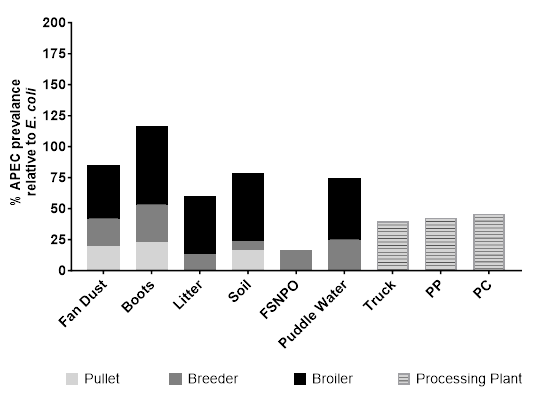


**45.8%**

**42.7%**

**39.9%**

**7.1%**

**22.5%**

**25%**

**16.6%**

**16.6%**

**12.9%**

**22.6%**

**19.4%**

**50%**

**55%**

**47%**

**63.5%**

**30.5%**

**43.2%**

**Supplementary Figure 2.** Prevalence of APEC relative to *E. coli*. APEC prevalence relative to *E. coli* across different farm types and sample types. FSNPO: fecal samples of non-poultry origin, PP: post-pick, PC: post-chill
